# Human umbilical cord mesenchymal stem cells restore the ovarian metabolome and rescue premature ovarian insufficiency in mice

**DOI:** 10.1186/s13287-020-01972-5

**Published:** 2020-11-04

**Authors:** Yan Zhao, Jiao Ma, Peiye Yi, Jun Wu, Feiyan Zhao, Wan Tu, Wenjing Liu, Tianda Li, Yan Deng, Jie Hao, Hongmei Wang, Long Yan

**Affiliations:** 1 State Key Laboratory of Stem Cell and Reproductive Biology, Institute of Zoology, Chinese Academy of Sciences, Beijing, 100101 China; 2 University of Chinese Academy of Sciences, Beijing, 100049 China; 3 Institute for Stem Cell and Regeneration, Chinese Academy of Sciences, Beijing, 100101 China; 4 National Stem Cell Resource Center, Chinese Academy of Sciences, Beijing, 100101 China; 5 Department of Human Reproductive Medicine, Beijing Obstetrics and Gynecology Hospital, Capital Medical University, Beijing, 100026 China

**Keywords:** Ovarian function restoration, Premature ovarian insufficiency, Mesenchymal stem cell, Metabolomics, Metabolite

## Abstract

**Background:**

Premature ovarian insufficiency (POI) is an ovarian dysfunction that seriously affects a woman’s physiological health and reproduction. Mesenchymal stem cell (MSC) transplantation offers a promising treatment option for ovarian restoration in rodent POI models. However, the efficacy and mechanism of it remain unclear.

**Methods:**

POI mice model was generated by cyclophosphamide and busulfan, followed with the treatment of tail-vein injection of the human umbilical cord mesenchymal stem cells (hUCMSCs). Maternal physiological changes and offspring behavior were detected. To reveal the pathogenesis and therapeutic mechanisms of POI, we first compared the metabolite profiles of healthy and POI ovarian tissues using untargeted metabolomics analyses. After stem cell therapy, we then collected the ovaries from control, POI, and hUCMSC-treated POI groups for lipid metabolomics and pseudotargeted metabolomics analysis.

**Results:**

Our results revealed remarkable changes of multiple metabolites, especially lipids, in ovarian tissues after POI generation. Following the transplantation of clinical-grade hUCMSCs, POI mice exhibited significant improvements in body weight, sex hormone levels, estrous cycles, and reproductive capacity. Lipid metabolomics and pseudotargeted metabolomics analyses for the ovaries showed that the metabolite levels in the POI group, mainly lipids, glycerophospholipids, steroids, and amino acids changed significantly compared with the controls’, and most of them returned to near-healthy levels after receiving hUCMSC treatment. Meanwhile, we also observed an increase of monosaccharide levels in the ovaries from POI mice and a decrease after stem cell treatment.

**Conclusions:**

hUCMSCs restore ovarian function through activating the PI3K pathway by promoting the level of free amino acids, consequently improving lipid metabolism and reducing the concentration of monosaccharides. These findings provide potential targets for the clinical diagnosis and treatment of POI.

## Background

Premature ovarian insufficiency (POI) is a defect characterized by the cessation of ovarian function before 40 years of age. In the general population, 1–2% of women experience POI [1–3] with consequent primary amenorrhea, secondary amenorrhea, premature sex steroid deficiency, infertility, and menopausal syndrome [3–7]. Currently, hormonal replacement treatment (HRT) remains the most widely used method for POI treatment. However, even long-term HRT can only partially suppress the disease while increasing the risk of cancer [4, 6–10].

Mesenchymal stem cell (MSC) transplantation has emerged as an validated and useful treatment option for regenerative medicine in recent years. Based on their differentiation potential and secretion capacity, MSCs have been widely used to treat multiple conditions, such as nerve diseases and cardiac diseases [11–16]. Recently, several studies have reported that MSC transplantation can also help restore ovarian function in rodent models, and these studies have tried to determine the underlying mechanisms by means of transcriptomic data analysis [17–23]. However, there is still a gap between transcriptomic information and the final biological phenotype.

Metabolomics is a bioanalytical strategy that provides information on the metabolite profiles of biological processes. It can be applied to identify metabolites in samples under normal conditions compared with disease-induced altered states and is considered as a powerful phenotyping tool [24]. Early studies have validated the reliability of metabolic changes for assessing oocyte quality and studying ovarian diseases, such as polycystic ovary syndrome (PCOS) [25–27], suggesting that metabolic variation may offer a suitable strategy for POI study.

In this study, we established a POI mouse model by intraperitoneally injecting cyclophosphamide and busulfan [20, 28, 29]. Ten days after POI induction, we transplanted GFP-labeled hUCMSCs by tail-vein injection to treat the POI mice. After treatment, we tracked hUCMSCs in the blood, the whole body, and the ovarian tissues to determine the extent of hUCMSC survival. We then systematically examined ovarian physiological indexes, including hormone levels, ovarian histological morphology, and reproductive capacity among normal, POI, and hUCMSC-treated POI mice. Subsequently, we employed untargeted metabolomics and identified metabolites that were significantly changed during POI induction and after stem cell treatment. Further lipid metabolism and pseudotargeted metabolomics analysis revealed a reversion of these metabolites after hUCMSC therapy. Moreover, through the analysis of metabolic pathways and related metabolites, we identified the potential molecular mechanism for the recovery of damaged ovaries.

## Methods

### Experimental animals

All animal procedures were conducted in accordance with the guidelines of the Institute of Zoology, Chinese Academy of Sciences, for the care and use of laboratory animals. Six-week-old female ICR mice were used in all experiments described below. After being purchased from SPF (Beijing) Biotechnology Company, the mice were fed a standard pellet diet and given free access to water. Vaginal smears were obtained daily, and only those who showed at least two consecutive normal 4 to 5-day estrous cycles by vaginal smears were included in the experiments.

### Animal model establishment

To establish the chemotherapy-induced POI model, 6-week-old female ICR mice were administered 120 mg/kg cyclophosphamide (CTX, Sigma, C0768) and 30 mg/kg Busulfan (BUS, Sigma, B2635) via intraperitoneal injection.

### hUCMSC culture and characterization

The hUCMSCs were kindly received from the National Stem Cell Resource Bank and cultured in serum-free medium as described in our previous study [30]. All the cells were used in accordance with standard experimental protocols approved by the Ethics Committee of the Institute of Zoology, Chinese Academy of Sciences.

### hUCMSC transplantation

Mice were randomly divided into three equal groups. The control group consisted of normal control mice with no treatment. In the POI group, the mice were administered CTX and BUS on day 0, as previously described. In the intravenous group, POI mice were intravenously injected with 1 × 10^6^ hUCMSCs in a volume of 0.1 ml of 0.1 M DPBS (pH 7.4, Gibco, 14040133) on day 10 (as illustrated in Fig. 4a).

### Body and ovarian weight

To quantify the differences among normal, POI-induced and hUCMSC-treated mice, their body and ovarian weights were measured with an analytical balance.

### Counting of ovarian follicles

Ten days after the hUCMSC treatment, the ovaries were collected, and the follicle numbers were counted. Fresh ovarian samples were fixed in 4% paraformaldehyde (Sigma, P6148) for at least 12 h. After dehydration and paraffin embedding, the samples were serially sectioned at 5 μm and then mounted on every fifth section. Routine hematoxylin (Solarbio, G1080-100) and eosin (ZSGB-BIO, ZLI-9613) (H&E) staining were performed for further histologic examination. Primordial, primary, secondary, and antral follicles were classified and counted. To avoid counting any follicle repeatedly, only those with an oocyte were included for further analysis.

### Cell tracking studies

GFP-positive hUCMSCs were kindly received from the National Stem Cell Resource Bank. For cell-tracking studies, flow cytometry, animal imaging, and the detection of GFP signals were performed as follows. For flow cytometry, the venous blood was collected from the endocanthion of each mouse at 1, 4, 24, and 48 h after hUCMSC transplantation. After incubation with a whole-blood red blood cell lysis reagent for 30 min at room temperature, the cell suspension was washed with PBS and then applied for flow cytometric analysis. For GFP signal detection, the mice were sacrificed 7 days after hUCMSC transplantation. Ovarian samples were paraffin-embedded and sectioned as described above. The sections were then observed for GFP signals with a fluorescence microscope.

### E2 and FSH measurements

The venous blood was collected from the endocanthion when mice were at the diestrous stage. The blood samples were then left at room temperature for 60 min. Upon coagulation, the samples were centrifuged at 4000 rpm/min for 15 min at 4 °C. The supernatant was then collected and sent to the Beijing North Institute of Biological Technology (Beijing, People’s Republic of China) for serum FSH and E2 measurements.

### Estrous cycle analysis

After the vaginal introitus of the mouse was washed several times, physiological saline (JC, 039-13315) was collected and smeared onto glass slides. The slides were fixed with 95% ethanol (Aladdin, E111992), washed with distilled water, and then subjected to H&E staining. Cell types and their proportions were quantified under an optical microscope.

### Mouse superovulation

Three groups of mice were superovulated 10 days after hUCMSC transplantation. All these mice were intraperitoneally injected with 7.5 IU pregnant mare serum gonadotropin (PMSG, Ningbo second hormone factory, China, YMXQ-0001), followed by intraperitoneal injection of 7.5 IU human chorionic gonadotropin (HCG, Ningbo second hormone factory, China, JS0001) 48 h later. The oocytes were collected 12 to 16 h after HCG injection.

### Nontarget metabolomics analysis

#### Sample preparation

Ovarian tissues were collected and rapidly washed in cold PBS to remove as much blood as possible. Washed samples were then transferred to QSP tubes and ground for 4 min in 400 μL methanol at 4 °C at 50 Hz. Then, the samples were vortex-mixed and centrifuged at 14,000 *g* for 15 min at 4 °C. Each 400 μL of supernatant was transferred into a new tube, lyophilized, and stored at − 20 °C.

#### Sample detection

Each tube of lyophilized sample was dissolved in 100 μL of 20% methanol solution. Ultra-performance liquid chromatography (UPLC) coupled to mass spectrometry (MS) was carried out. All measurements were collected by Xcalibur data acquisition software (Thermo, USA). One blank sample (20% methanol/aqueous solution) and two quality control (QC) samples (generated by combining a portion of each sample, with the same preparation and data acquisition methods) were also added to the plate for analysis.

#### Data acquisition

Ionization of metabolites was carried out by using an electrospray ionization (ESI) source in both positive and negative modes, and data were collected simultaneously in the two modes. The mass spectrometry parameters were as follows: the mass range (m/z) was from 75 to 1100; the spray voltages were 3.50 and 2.50 kV in the two modes, respectively; the capillary temperature was 320 °C; and the flow rates of the sheath and auxiliary gas were 40 and 10 arbitrary units (Arb), respectively. The resolution was 70,000 in full-scan mode, the automatic gain control (AGC) target was a 3 × 10^6^ ion capacity, and the maximum injection time (IT) was 200 ms. For full MS/dd-MS2 analysis, the parameters of resolution, AGC target, maximum TT, and NCE were 17,500, 1 × 10^5^, 50 ms, and 20 and 40 eV, respectively.

### Pseudotargeted metabolomics analysis

#### Sample preparation and detection

The same method was used for nontarget metabolomics. Selection of the target ion pair of samples by the method described above and then the model of mass spectrometry multiple reaction monitoring (MRM) or parallel reaction monitoring (PRM) were used for analysis, based on the information of these ion pairs, to collect the pseudotargeted data.

#### Data acquisition

Ionization of metabolites was carried out by using an ESI source in both positive and negative modes, and data were collected simultaneously in the two modes. The mass spectrometry parameters were as follows: the mass range (m/z) was from 100 to 1100; the spray voltages were 3.80 and 3.00 kV in the two modes, respectively; the capillary temperature was 320 °C; and the flow rates of the sheath and auxiliary gas were 35 and 8 Arb, respectively. The resolution was 70,000 in full-scan mode. For full MS/dd-MS2 analysis, the parameters of resolution and NCE were 17,500 and 20 and 40 eV, respectively.

### Lipidomics analysis

#### Sample preparation

Ovarian tissues were collected and rapidly washed in cold PBS to remove as much blood as possible. Washed samples were then transferred to QSP tubes and ground for 4 min in 400 μL of methanol at 4 °C at 50 Hz. Then, 1 mL of methyltert-butyl ether (MTBE) was added, the sample was mixed by vortex for 1 min, and the sample was shaken for 1 h at room temperature. Then, 250 μL of water was added, the sample was vortexed for 1 min, and the sample was allowed to stand for 1 h at room temperature. Samples were centrifuged at 14000 *g* for 15 min at 4 °C. Each 400 μL of supernatant was transferred into a new tube, lyophilized, and stored at − 20 °C.

#### Sample detection

The same as we described in nontarget metabolomics.

#### Data acquisition

Ionization of metabolites was carried out by using an ESI source in both positive and negative modes, and data were collected simultaneously in the two modes. The mass spectrometry parameters were as follows: the mass range (m/z) was from 100 to 1500; the spray voltages were 3.50 and 3.00 kV in the two modes, respectively; the capillary temperature was 350 °C, and the flow rates of the sheath and auxiliary gas were 45 and 10 Arb, respectively. The resolution was 70,000 in full-scan mode. For full MS/dd-MS2 analysis, the parameters of resolution and NCE were 17,500 and 25, 35, and 45 eV, respectively.

### Statistical analysis

The statistical analyses were performed using SPSS 21.0. One-way ANOVA with LSD tests was used to determine significant differences among the three groups. A *P* value less than 0.05 was considered to be statistically significant. All data are presented as the mean ± SD.

## Results

### Characteristics of chemotherapy-induced POI mice

**Fig. 1:**
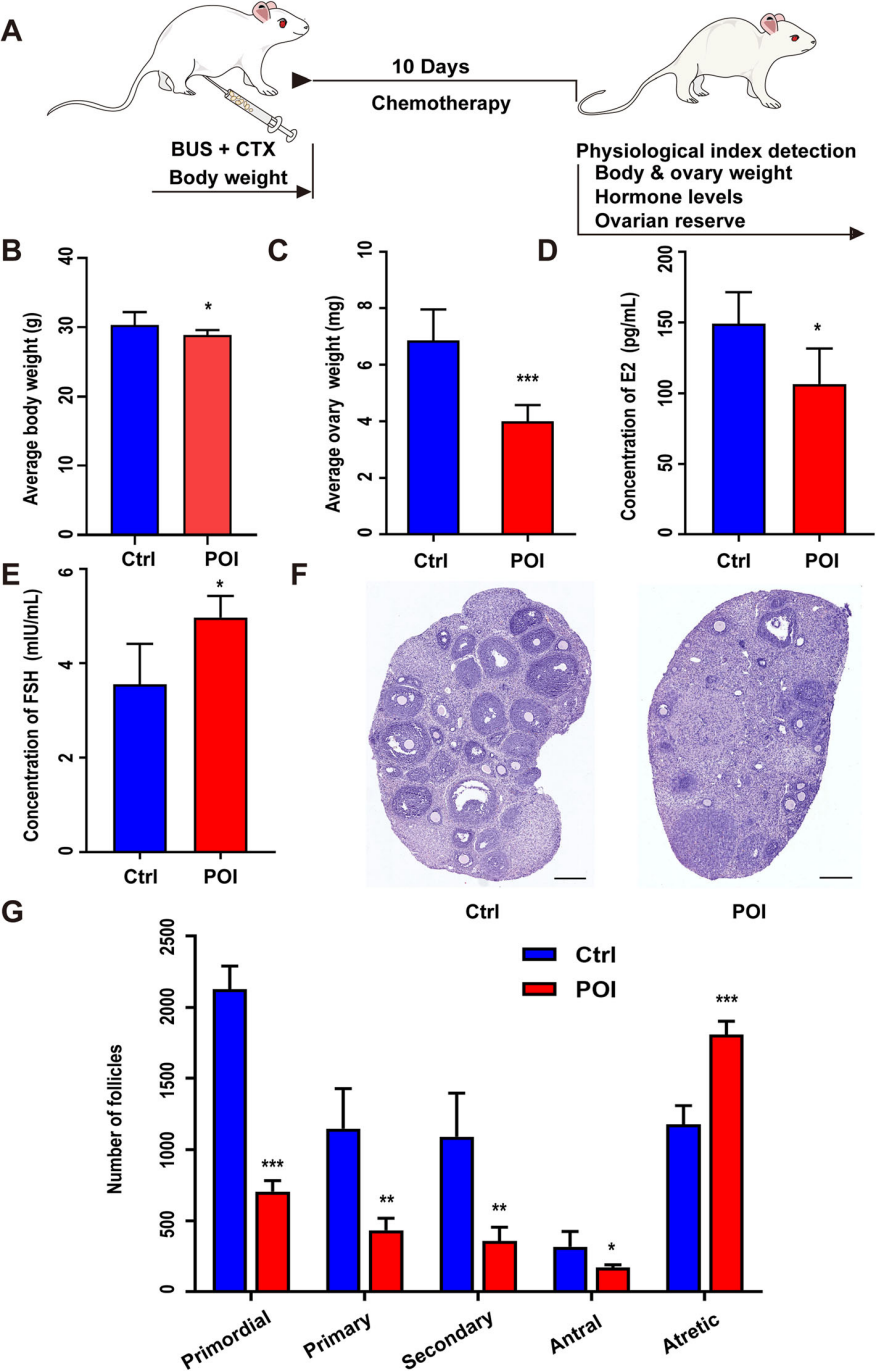
Establishment of the mouse POI model. **a** Schematic procedure of establishing the chemotherapy-induced POI model. **b** The comparison of body weight between the control and POI groups (*n* = 10). **c** The comparison of ovarian weight between the control and POI groups (*n* = 17). **d**, **e** The comparison of serum levels of FSH and E2 between the control and POI groups (*n* = 4). **f** H&E staining of ovarian sections from the control and POI groups. **g** The number of follicles at different developmental stages from the control and POI groups. Scale bar = 400 μm. All data were presented as the mean ± SD. One-way ANOVA with LSD and Tamhane’s T2 post hoc test were used for analysis. *P < 0.05; **P < 0.01; ***P < 0.001

We first created a chemically induced POI mouse model by injecting cyclophosphamide and busulfan. To ensure the successful modeling of POI, we assessed several specific physiological indexes 10 days after the treatment of chemotherapeutics (Fig. 1a). Both body weight and ovarian weight were significantly lower in the POI mice than in the healthy controls (Fig. 1b, c). Plasma samples were then collected to measure the concentrations of FSH and E2 in both the control and POI groups. In the POI mice, the levels of E2 significantly decreased, and the levels of FSH increased obviously (Fig. 1d, e). We also carried out pathological studies to evaluate the ovarian reserve of each group and found that compared to those in the control group, the ovaries in the chemotherapy group appeared with more atrophic but fewer developing follicles (Fig. 1f). Subsequently, we analyzed the number of follicles at all stages and confirmed a significant reduction in primordial, primary, secondary, and antral follicles and a dramatic increase in atretic follicles after chemotherapy (Fig. 1g).

### Metabolic differences between POI and control mice

To determine the pathogenesis of POI, we collected ovarian tissues (control = 29, POI = 29) and serum samples (control = 28, POI = 30) and compared their metabolic profiles using liquid chromatography-mass spectrometry (LC-MS). The total ion chromatogram (TIC) and basic peak ion chromatogram (BPI) revealed that the majority of metabolites were eluted during the first 25 min, indicating that these data qualified for further analyses (Figs. S1A, S2A). Among the QC samples, more than 60% in the ovarian tissues (Fig. S1B) and 80% in the serum group (data not shown) had mass spectrometric characteristics with relative standard deviations (RSDs) < 30%.

**Fig. 2:**
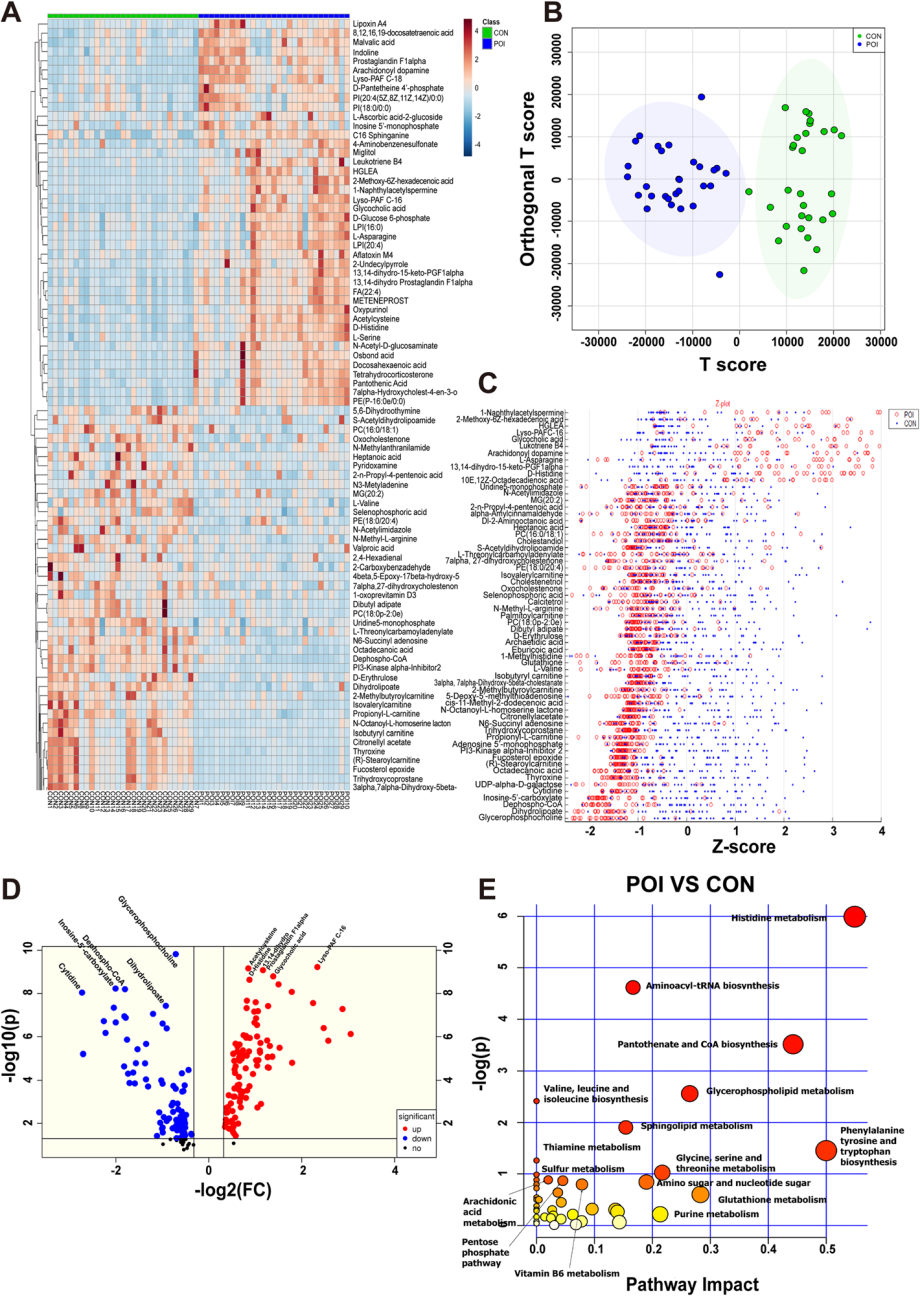
Metabolomics analysis of ovarian tissues from the control and POI groups. **a** Heat map analysis of differential metabolites changes induced by chemotherapy in ovarian samples (control and POI groups). **b** OPLS-DA score plot of ovarian samples from both the control and POI groups. **c** *Z*-score plot of the screened metabolites in the ovarian samples of the control group and POI group. Each spot represents a metabolite. The control group is shown in blue, and the POI group is shown in red. *Z*-scores are sorted in descending order, in which the magnitude of the change indicates the fold by which the mean of the control group is higher or lower than its standard deviation. **d** Volcano plot of screened differential metabolites for both groups. Each spot represents a metabolite, and the scattered spots represent the final screening results. Significantly upregulated metabolites are shown in red, significantly downregulated metabolites are shown in blue, and nonsignificantly different metabolites are shown in black. **e** KEGG was used to analyze the metabolic pathways in ovarian tissues, and metabolic pathways with *P* < 0.05 are shown

Metabolites that differentially expressed between the two groups were analyzed for underlying mechanisms. A heat map revealed that the levels of a large variety of metabolites changed after chemotherapy in both the ovarian tissue and serum samples (Figs. 2A, S2B). We established a relationship model between metabolite levels and the loaded samples using orthogonal partial least squares discrimination analysis (OPLS-DA) (Figs. 2B, S2C). The overall levels of metabolites were significantly different between the control and POI samples, suggesting the high accuracy of the model prediction (ovarian samples, *R*^2^ = 0.70, *Q*^2^ = 0.64; serum samples, *R*^2^ = 0.84, *Q*^2^ = 0.74) (Figs. S1C, S2D). Based on their *P* values, fold changes, and variable importance in projection scores (*P* < 0.05, FC > 1.5, VIP > 1), 1190 features were determined. In further qualitative analysis, 180 different metabolites were identified in ovarian samples, among which 99 were increased and 81 were decreased (Fig. 2c, d, Table S1). Furthermore, 297 metabolites were identified in serum samples, among which 148 were increased and 149 were decreased (Fig. S2E, Table S2). Many of these metabolites, including PC (e.g., PC (16:0/18:0), PC (16:0/18:1), LysoPC (20:4), LysoPC (18:1)), PE (e.g., LysoPE (20:3), LysoPE (20:4), PE (18:0/22:4)), and carnitine (e.g., (R)-stearoylcarnitine, 2-methylbutyroylcarnitine, and isobutyryl carnitine) were lipid metabolites, suggesting that the POI pathogenesis might be highly associated with abnormal lipid metabolism. Other metabolites were involved in the metabolism of amino acids, especially histidine, tyrosine, tryptophan, and serine (Fig. 2c, d). Pathway analyses further revealed that many indicators related to lipid metabolism (such as glycerophospholipid metabolism and sphingolipid metabolism) and amino acid metabolism (including histidine metabolism and aminoacyl-tRNA biosynthesis) were remarkably changed in ovarian tissues after POI induction (Fig. 2e). In the serum samples, many of the differential metabolites were related to amino acid metabolism, but their fold changes were not as notable as those of the ovarian tissues (Fig. S2F).

### Establishment of clinical-grade hUCMSCs and in vivo tracing

**Fig. 3:**
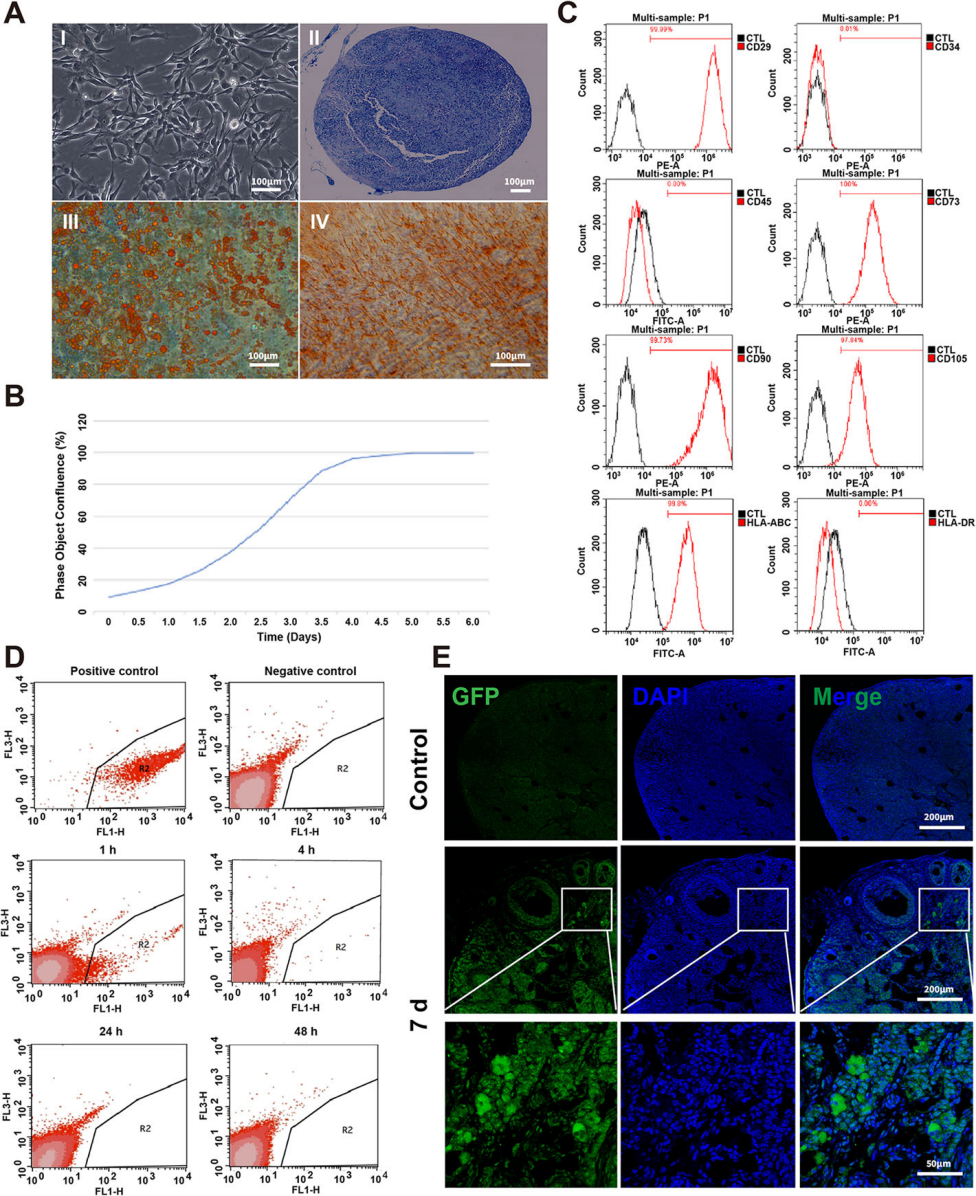
Identification of clinical-grade hUCMSCs and in vivo tracing. **a** Cultured hUCMSCs exhibited typical fibroblastic morphology (I) and could differentiate into chondroblasts (II), lipoblasts (III), and osteoblasts (IV). **b** Growth curve of hUCMSCs. **c** Flow cytometric analysis of hUCMSCs showed their expression of CD29^+^ CD73^+^ CD90^+^ CD105^+^ CD45^−^ CD34^−^. **d** After intravenous transplantation for 1 h, 4 h, 24 h, and 48 h, DIR dye-labeled hUCMSCs in peripheral blood cells (the red blood cells had been dissolved) were analyzed by flow cytometry. The peripheral blood cells (the red blood cells had been dissolved) in POI mice that received only DPBS were used as the negative control. DIR dye-labeled hUCMSCs were used as the positive control. **e** GFP-labeled hUCMSCs in the ovaries were detected by immunofluorescence after transplantation

Clinical grade hUCMSCs were established according to the good manufacturing practice (GMP) of medical product standards. The hUCMSCs resembled fibroblasts morphologically; had the ability to differentiate into chondroblasts, lipoblasts, and osteoblasts (Fig. 3a); and exhibited a normal growth rate during expansion in vitro (Fig. 3b). The hUCMSCs were positive for classical MSC markers and negative for hematopoietic markers, in accordance with the International Society for Cellular Therapy (ISCT) criteria (Fig. 3c). Subsequently, we stained hUCMSCs with DiR and injected them into chemically induced POI mice. Fluorescent DiR signals were visible 1 h after hUCMSC transplantation but fell below the detection limit 24 h after transplantation (Fig. 3d). To definitively validate the engraftment or rejection of hUCMSCs after injection, we transplanted GFP-labeled hUCMSCs via intravenous injection, and an immunofluorescence analysis verified the survival of engrafted hUCMSCs in recipient ovarian tissues at day 7 post-transplantation (Fig. 3e).

### hUCMSC transplantation restored ovarian function in POI mice

**Fig. 4:**
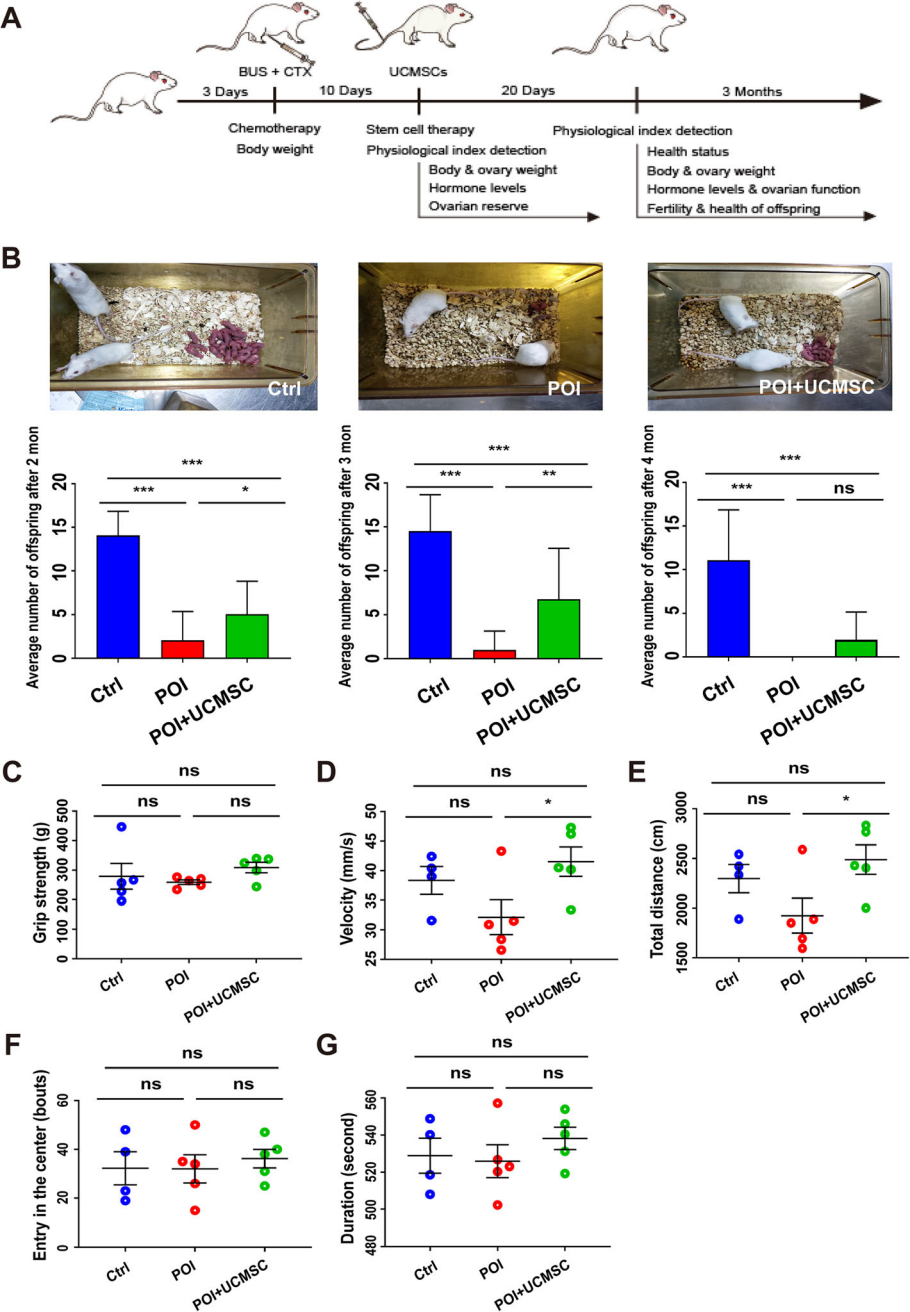
The offspring of POI mice increased in number after hUCMSC transplantation and manifested normal behavior. **a** Schematic procedure of hUCMSC treatment. **b** Fertility test of three groups of mice. The test was performed at 1, 2, and 3 months after hUCMSC transplantation. The number of offspring per liter was analyzed (*n* = 15, *n* = 15, *n* = 15). Behavioral tests of the offspring in the three groups (*n* = 4, *n* = 5, *n* = 5) include **c** grip strength, **d** velocity, **e** total distance, **f** length of duration, and **g** times of entry into the center. All data were presented as the mean ± SD. One-way ANOVA with LSD and Tamhane’s T2 post hoc test were used for analysis. *P < 0.05; **P < 0.01; ***P < 0.001

To evaluate the efficacy of hUCMSC treatment, we systematically evaluated their physiological indices 20 days after transplantation, including body weight, ovarian weight, hormone levels, estrous cycles, ovarian structure, and follicular reserves (Fig. 4a). In the hUCMSC-treated POI mice, all of these indices partially recovered to some extent (Fig. S3, S4). To further assess hUCMSC-mediated fertility restoration, female mice from all three groups were mated with proven fertile males. Two months after hUCMSC treatment, the total offspring numbers of the treated POI mice were significantly higher than those of the untreated POI mice (5.07 ± 3.64 versus 2.07 ± 3.19), indicating restoration of the ovarian function (Fig. 4b). To determine whether these treatments influence offspring health, we evaluated the behavior of the offspring via several physical indices (grip strength, velocity, total distance, entry into the center, and duration) (Fig. 4c–g). Most of the physical indices did not show significant differences between the hUCMSC-treated POI and control groups, although the running velocity and running distance of the offspring from the hUCMSC-treated POI group were significantly better than those of the offspring from the POI group (Fig. 4d, e), demonstrating that the offspring of hUCMSC-treated POI mice were as healthy as those of the control mice.

### Untargeted lipid metabolomics revealed that small-molecule lipids play an important role in the recovery of ovarian function

After the validation of POI modeling and hUCMSC treatment, we collected ovarian tissues from the control, POI, and hUCMSC-treated POI mice and tried to clarify the mechanisms by which hUCMSCs promoted ovarian restoration.

**Fig. 5:**
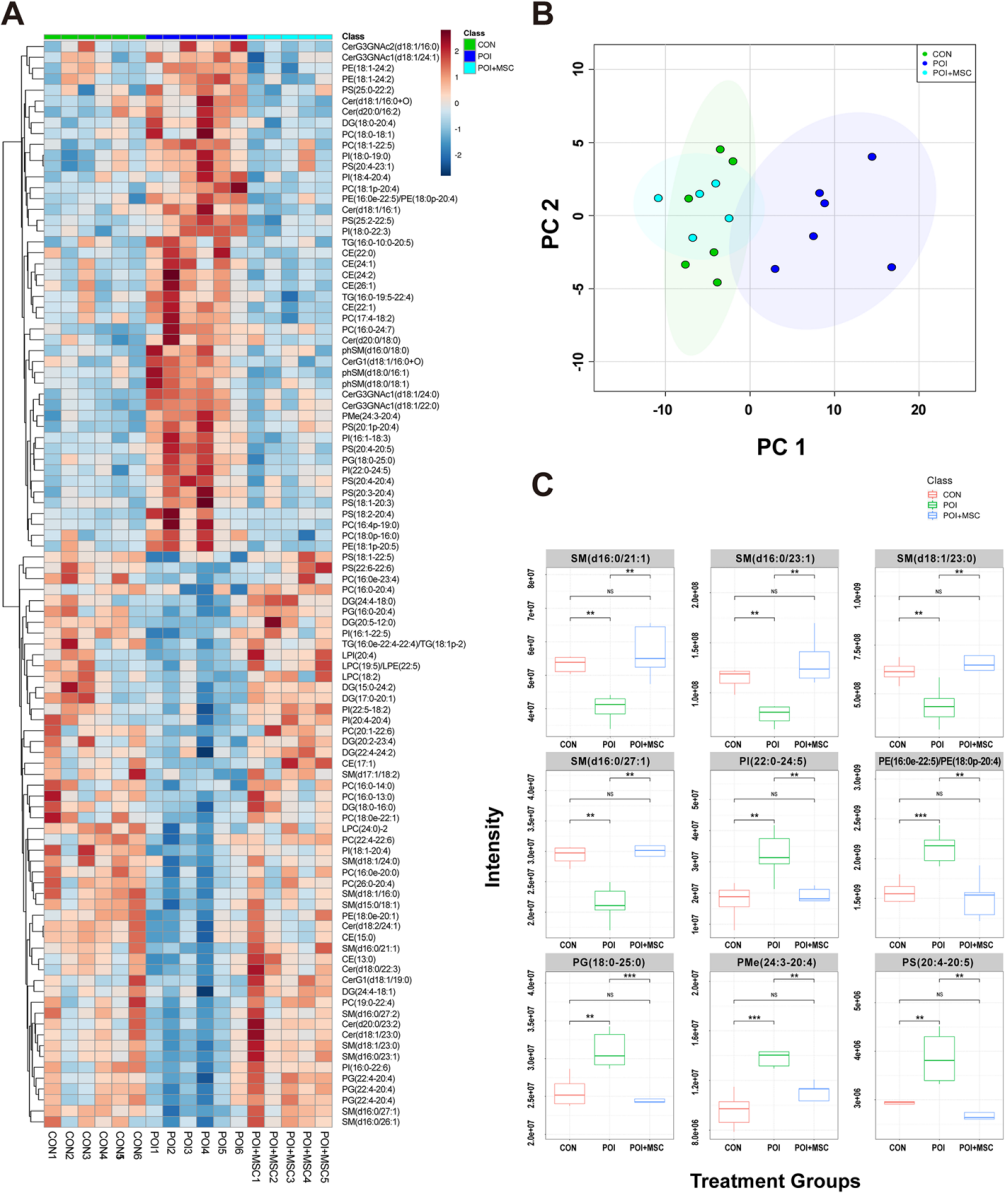
Nontarget metabolomics of lipids from ovarian samples. **a** Heat map analysis of three ovarian samples (control, POI, and POI + hUCMSCs), which shows the changes in the expression of different metabolites after treatment. **b** PCA score plot of the ovarian samples of all three groups. **c** The box plot shows the changes in representative metabolites after hUCMSC treatment

According to previous metabolic analyses, POI was highly associated with abnormal lipid metabolism in ovarian tissues (Fig. 2). Therefore, we employed untargeted lipidomics to clarify the lipid diversity among the three groups. Heat map and principal component analysis (PCA) showed that lipidomic patterns changed remarkably after POI modeling, but many of them recovered to normal levels after hUCMSC transplantation (Fig. 5a, b, Fig. S5A, D). The abundance of 120 lipid metabolites became significantly altered after chemical injection and POI induction, and 95 of these metabolites returned to near-normal levels after hUCMSC treatment (Table S3). For example, the levels of SM (d16:0/21:1), SM (d16:0/23:1), SM (d18:1/23:0), SM (d16:0/27:1), and PI (22:0-24:5) decreased after chemotherapy and recovered after hUCMSC transplantation, while the levels of PE (16:0e-22:5)/PE (18:0p-20:4), PG (18:0-25:0), PMe (24:3-20:4), and PS (20:4-20:5) were elevated after chemotherapy and then dropped to normal levels after hUCMSC injection (Fig. 5c and Fig. S5B-C, E-F).

### Pseudotargeted metabolomics revealed an association between ovarian functional recovery and targeted metabolites

**Fig. 6:**
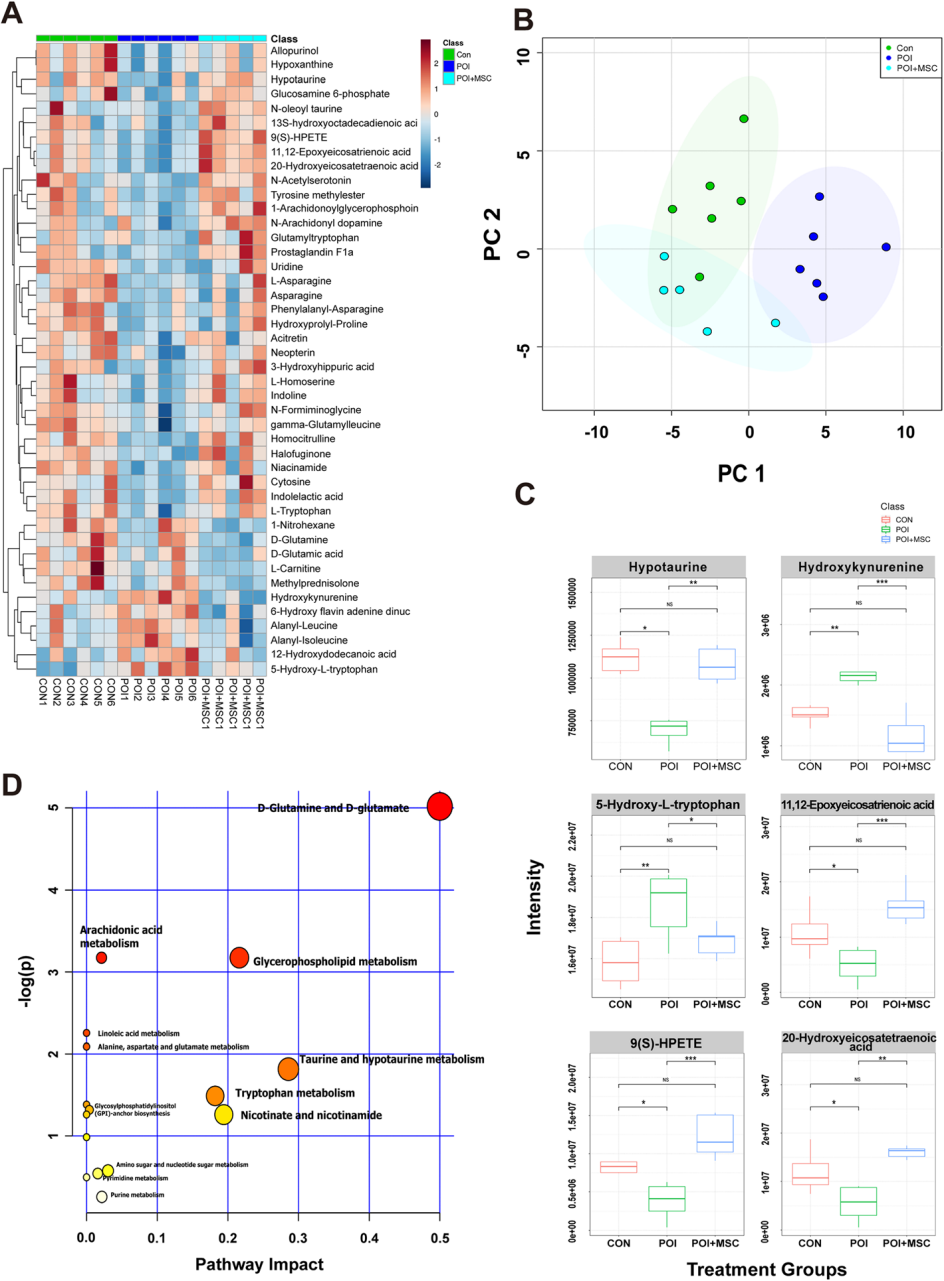
Pseudotargeted metabolomics of nonlipids from ovarian samples. **a** Heat map of all three groups shows the trends of differential metabolite expression pattern before and after the hUCMSC treatment. **b** PCA score plot reveals differences in the expression levels of targeted metabolites after hUCMSC transplantation. **c** The box chart shows that pseudotargeted metabolomics is designed for the differential metabolites screened after POI modeling. The results show that the expression of some metabolites returns to the level of the control group after treatment. **d** KEGG was used to analyze the metabolic pathways of different metabolites that changed significantly after hUCMSC transplantation

In addition, we performed a pseudotargeted metabolic analysis to further evaluate the roles of 55 selected metabolites (excluding lipid metabolites) based on previous data (Fig. 2, Table S4). Heat map and PCA analysis showed that these metabolites changed remarkably after chemical induction but that most of them recovered to normal levels after hUCMSC transplantation (Fig. 6a, b and Fig. S6A, D). For instance, hypotaurine, 11,12-epoxyeicosatrienoic acid, 9(S)-HPETE, and 20-hydroxyeicosatetraenoic acid decreased after chemotherapy and recovered after hUCMSC treatment. Additionally, hydroxykynurenine and 5-hydroxy-l-tryptophan were elevated by chemotherapy and then declined after hUCMSC therapy (Fig. 6c, Fig. S6B-C, E-F). Pathway analysis revealed that these differentially expressed metabolites were mainly related to pathways involving d-glutamine and d-glutamate, glycerophospholipid metabolism, taurine, and hypotaurine metabolism (Fig. 6d).

### Mechanisms whereby hUCMSC therapy restores impaired ovarian function

**Fig. 7:**
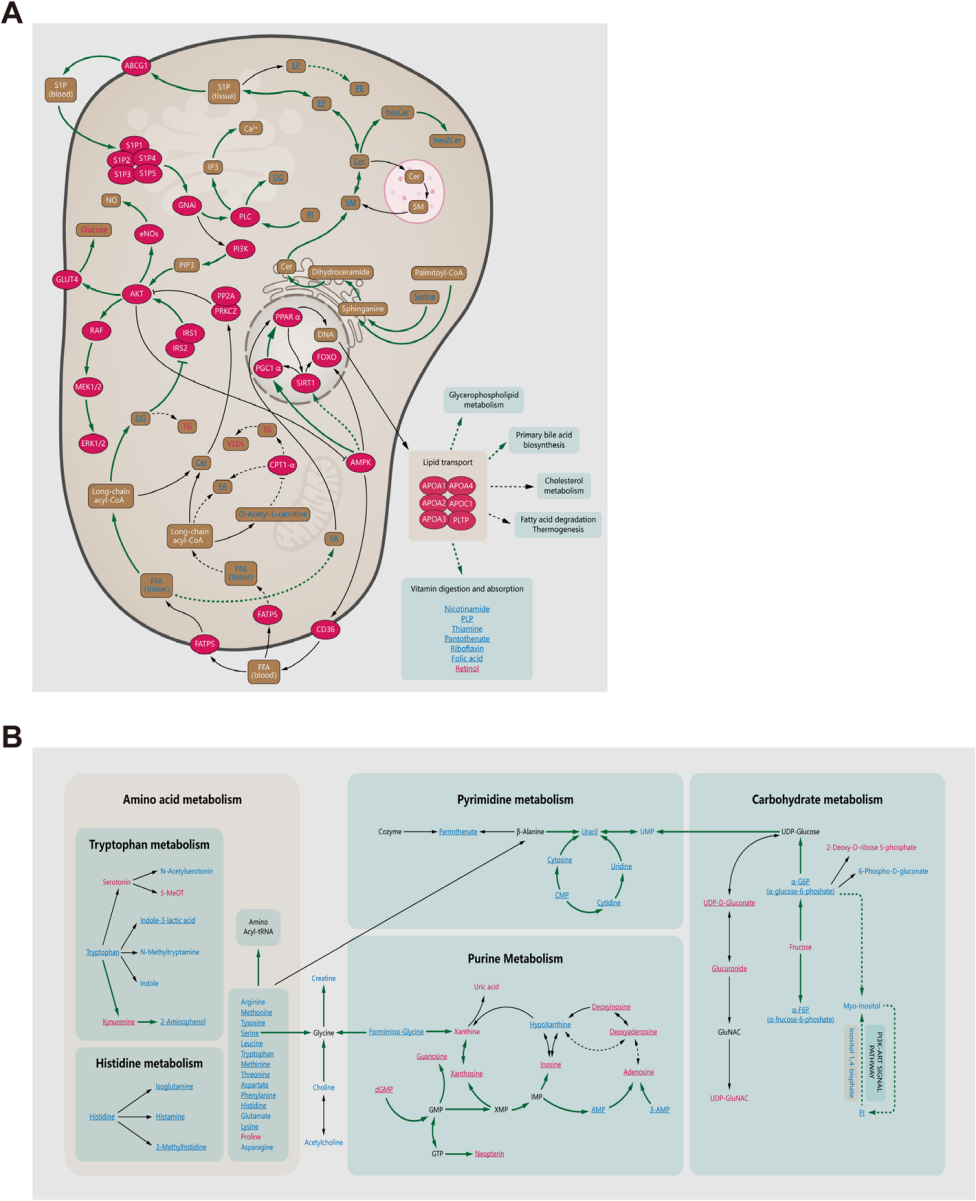
Metabolomics analysis of the mechanisms of hUCMSC treatment for POI. The pathway map shows the changes in metabolites and metabolic pathways in the ovaries of the hUCMSC-treated group compared to those of the POI group. **a** Map of regulation pathways involved in total metabolite. **b** Map of regulatory pathways between amino acids, purines, pyrimidines, and carbohydrate metabolism. Significantly upregulated metabolites are shown in red, significantly downregulated metabolites are shown in blue, and nonsignificant different metabolites are shown in black. The names in bold are the relevant metabolites detected in the nontarget/target metabolomics analysis. The substance in the red ellipse represents the enzyme or its corresponding protein. The solid arrow indicates that metabolites (enzymes/proteins and metabolites) have a direct relationship with each other, while the dotted arrow indicates a distant relationship. Tissues or parts of metabolic processes (such as lysosomes) are represented by solid frames, and related pathways (such as glycerolipid pathways) are represented by dotted frames. The underlined metabolites represent the metabolites that recovered after hUCMSC transplantation compared to the changes after POI induction. The green arrows represent the paths recovered. Abbreviations: Cer, *N*-acylsphingosine; DG, diacylglycerol; FA, fatty acid; FFA, free fatty acid; hexCer, glucosylceramide; hex2Cer, lactosylceramide; PE, phosphatidylethanolamine; PI, phosphatidyl-myo-Insitol; SM, sphingomyelin; phSM, sphingomyelin-1-phosphate; TG, triacylglycerol; α-G6P, α-glucose-6-phoshate; α-F6P, α-frucose-6-phoshate

We then tried to further summarize the mechanisms by which hUCMSCs promoted ovarian functional recovery in POI mice. According to untargeted lipidomic analysis, the major lipid pathways influenced by POI modeling included glycerophospholipid metabolism, sphingolipid metabolism, the PI3K-MAPK signaling pathway, lipid transport and related pathways (cholesterol metabolism, bile acid metabolism, lipid digestion, and absorption), and hormonal regulation (steroid hormone metabolism). After stem cell treatment, many of them, most notably glycerophospholipid metabolism and steroid hormone metabolism, recovered to near-normal levels, (Fig. 7a, Fig. S7A-B). Additionally, the pseudotargeted metabolomics approach revealed that some other metabolites might also play an important role in hUCMSC treatment by influencing amino acid metabolism, purine metabolism, pyrimidine metabolism, glycometabolism, and nitrogen metabolism. Ovarian dysfunction caused abnormal nitrogen metabolism, and the sharp decline in lipoamino acids in the ovaries led to insufficient tRNA synthesis, which on the one hand promoted purine metabolism and increased uric acid production and, on the other hand, inhibited pyrimidine metabolism. In addition, insufficient amino acids and lipid transport impeded the metabolism of glycolipids, which in turn increased the production of monosaccharides and glycolic acids. Thus, stem cell therapy effectively promoted an increase in amino acids to normal levels, regulated the recovery of lipid transport and metabolism, and balanced purine and pyrimidine metabolism within the ovarian tissues of POI mice (Fig. 7b).

## Discussion

As a potential therapeutic agent, the stem cells are limited in widely clinical application because of the security concern. In 2019, Zhao et al. assessed and reported the tumorigenicity of hUCMSCs at various passages. They found hUCMSCs have multiple mutation spectrum but without tumor formation capacity in vivo [31]. In this study, we made a long-term observation of mice treated with stem cell. All of the mice were in good health. They had normal behaviors and social ability, and no abnormalities were observed in their lives and reproductive activities. None of them died until the end of the trial. In addition, after dissection, all organs in hUCMSC-treated mice, including ovaries, were normal without neoplasia. In this study, we did not find any adverse effect from the mice with injection of hUCMSCs.

To date, some studies have indicated that MSCS transplantation can promote ovarian functional restoration in POI mice, as indicated by the recovery of body weight, ovarian weight, hormone levels, and ovarian reserve after stem cell therapy [21, 23, 32–43]. However, the mechanisms by which MSCs exert their actions have not been fully characterized. Several studies have tried to elucidate these mechanisms by RNA-Seq technology. Xia et al. discovered that cocultured MSCs supported human follicle survival and development by upregulating the expression of growth differentiation factor 9 (GDF9) and bone morphogenetic protein 15 (BMP15) [44]. In our previous study, we demonstrated that the apoptosis of ovarian cells was inhibited and that ovarian function was protected in MSCS conditional medium, owing to the upregulation of colony-stimulating factors (most notably glial cell line-derived neurotrophic factor (GDNF) and brain-derived neurotrophic factor (BDNF) and the activation of the PI3K pathway) [45]. In contrast to transcriptomics, metabolites reveal the interface between the encoded genomic information and the functional phenotype; thus, metabolomics can be used as a more powerful phenotyping tool for disease research. Therefore, we used metabolomics to explore the mechanism of action of hUCMSC treatment for POI.

As reported by Maidarti and colleagues, the PI3K/Akt pathway is critical for ovarian development, follicular growth, and the proliferation and differentiation of granulosa cells [46]. Sphingosine (such as S1P, Fig. 7a) is an important signal molecule that activates the PI3K pathway [47, 48], and serine is an essential ingredient in the synthesis of sphingolipids [49]. Our data indicated a significant reduction in serine in POI ovary, which could downregulate the production of sphingolipids and further inhibit the PI3K/Akt pathway. PI3K regulates the intracellular vesicle traffic [50], an important mode of lipid transportation. Inhibition of the PI3K pathway further limits lipid transportation and restricts the synthesis of steroid hormones (Fig. S7B), which in turn causes deterioration of the ovarian environment and POI. Through stem cell therapy, the restoration of the PI3K pathway improves the transport capacity of lipid metabolites and promotes the synthesis and secretion of hormones, leading to the recovery of ovarian function. On the other hand, the inhibition of the PI3K pathway affects glycometabolism, resulting in an increase in monosaccharides (Fig. 7b); increased monosaccharide levels are associated with apoptosis [51]. Likewise, our results demonstrated a significant increase in monosaccharides (Fig. 7b). To date, we have advanced a hypothesis that hUCMSCs restore ovarian function through activating the PI3K pathway by promoting the level of free amino acids, consequently improving lipid metabolism and reducing the concentration of monosaccharides.

In addition, intravenously injected stem cells may have other influences on the whole body. In the current study, we also compared metabolite changes in serum samples. Although their fold changes were not as notable as those in the ovarian tissues, some of them might also play a role in other important metabolic processes (such as glycerophospholipid metabolism, pyruvate metabolism, thiamine metabolism, and tryptophan metabolism). Further analysis of the serum data may help us unveil more details of how hUCMSCs promote ovarian restoration by regulating metabolism in the whole body.

Moreover, there are no previous studies of POI treatment focusing on the health of offspring. Therefore, we examined the behavior of the offspring of the control, POI, and hUCMSC-treated groups. The results of physiological tests demonstrated that the behaviors of the offspring from the hUCMSC-treated group were identical to the normal behaviors of the offspring from the control group, indicating that stem cell therapy has no adverse effect on offspring. We also found that there was no significant difference between the POI group and control group in terms of offspring behavior, which demonstrated that even though chemotherapy can weaken the fertility of the females, it does not influence the viability of their offspring.

## Conclusions

In summary, mesenchymal stem cell transplantation is effective in treating POI mice. Metabolomics studies manifested that hUCMSCs restore ovarian function through activating the PI3K pathway by promoting the level of free amino acids, consequently improving lipid metabolism and reducing the concentration of monosaccharides. To the best of our knowledge, this is the first study to reveal metabolite diversity among healthy, POI, and clinical grade hUCMSC-treated POI mouse ovaries. Our findings provide us with better knowledge of POI pathogenic mechanisms and may shed light on potential targets for its clinical diagnosis and the possible clinical translation of MSCs.

## Supplementary information

**Additional file 1: Supplementary figure 1.** Metabolomics-data quality analysis of tissue samples from the control and POI modeling groups. **A** Total ion chromatograms (TICs) of all ovarian samples (the sum of the total number of ions and their time-varying curves for the various specific charges), which reflect the overall information of the samples. **B** Analysis of characteristic differences in quality control (QC) samples by metabolic mass spectrometry. The abscissa represents the relative standard deviation (RSD) of metabolic characteristics in QC samples, and the ordinate represents the percentage of metabolic characteristics whose RSD falls within the corresponding range. **C** Orthogonal partial least squares discriminatory analysis (OPLS-DA) was used to establish a model of the relationship between the expression of metabolites and samples, and the goodness of fit of the model could be predicted by the values of R^2^ and Q^2^. R^2^=0.70, Q^2^=0.64.

**Additional file 2: Supplementary figure 2.** Metabolomics analysis of blood samples from the control group and POI model group. **A** Total ion chromatograms (TICs) of all blood samples, the sum of the total number of ions and their time-varying curves for the various specific charges, which reflect the overall information of the samples. **B** Heat map analysis of differential metabolome changes induced by chemotherapy in blood samples (control and POI groups). **C** OPLS-DA score plot of blood samples from both the control and POI groups. **D** Orthogonal partial least squares discriminatory analysis (OPLS-DA) was used to establish a model of the relationship between the expression of metabolites and blood samples, and the goodness of fit of the model could be predicted by the values of R2 and Q2. R2=0.84, Q2=0.74. **E** Volcano plot of screened differential metabolites in both groups. Each spot represents a metabolite, and the scattered spots represent the final screening result. Significantly upregulated metabolites are shown in red, significantly downregulated metabolites are shown in blue, and non-significantly different metabolites are shown in black. **F** KEGG was used to analyze the metabolic pathways in blood samples, and metabolic pathways with *P* < 0.05 are shown.

**Additional file 3: Supplementary figure 3.** hUCMSCs transplantation promotes ovarian health status. **A** Line chart of body weight changes in the three groups of mice within 4 weeks after hUCMSCs transplantation (*n*=6, *n*=9, *n*=9). **B** Photograph of ovaries removed from the three groups after the hUCMSCs treatment. **C** The weights of the ovaries among the three groups were compared after the hUCMSCs treatment. **D** Measurement of serum E2 levels after hUCMSCs transplantation (*n*=4). **E** Measurement of serum FSH levels after hUCMSCs transplantation (*n*=4). **F** Immunohistochemical analysis of AMH expression (red arrowhead) in mouse ovaries. Scale bar = 500 μm. **G** The line charts are representative of estrous cycles in the three groups detected after the treatment cycle. D: diestrus, M: metestrus, E: estrus, P: proestrus. **H** The percentage of mice whose estrous cycles returned to normal after the treatment cycle (*n*=8, *n*=9, *n*=10). All data were presented as the mean ± SD. One-way ANOVA with LSD and Tamhane’s T2 post hoc test were used for analysis. *p < 0.05; **p < 0.01; ***p < 0.001.

**Additional file 4: Supplementary figure 4.** hUCMSCs transplantation promotes ovarian functional reserve. **A** Representative oocytes of three groups after superovulation. Scale bar = 400 μm. **B** H&E staining of ovarian morphological changes after hUCMSCs transplantation. Scale bar = 400 μm. **C** Quantitative analysis of different categories of ovarian follicles after hUCMSCs transplantation (*n*=5, *n*=6, *n*=5). All data were presented as the mean ± SD. One-way ANOVA with LSD and Tamhane’s T2 post hoc test were used for analysis. *p < 0.05; **p < 0.01; ***p < 0.001.

**Additional file 5: Supplementary figure 5.** Nontarget metabolomics of lipids from ovarian samples. **A** Heat map showing that the expression levels of many lipid metabolites changed after POI modeling and that the patterns of the control and POI groups were significantly different. **B** Z-score plot of screened metabolites in the POI group with significant differences in the mean and standard deviation from that of the control group. Each spot represents a metabolite. The control group is shown in blue, and the POI group is shown in red. Z-scores were sorted in descending order, in which the magnitude of the change indicates the fold by which the mean of the control group is higher or lower than its standard deviation. **C** Volcano plot of the screened differential lipid metabolites measured in the control and POI groups. Each spot represents a metabolite, and the scattered spots represent the final screening result. Significantly upregulated metabolites are shown in red, significantly downregulated metabolites are shown in blue, and nonsignificant different metabolites are shown in black. **D** Heat map showing that the expression levels of a large number of metabolites from the POI+hUCMSCs group changed significantly compared with those from the POI group after transplantation. **E** Z-score plot of the metabolites screened in the samples of the POI+hUCMSCs group, with significant differences in the mean and standard deviation from those of the POI group. **F** Volcano plots of screened lipid metabolites measured in the POI and POI+hUCMSCs groups, with significant changes.

**Additional file 6: Supplementary figure 6.** Pseudotargeted metabolomics of ovarian tissue samples. **A** Heat map results show that the expression levels of a large number of metabolites changed significantly after POI induction, and most decreased. **B, C** Z-score plot and volcano plot show that the expression levels of the metabolites unrelated to lipids were significantly changed after POI induction, and most decreased. **D** Heatmap, **E** z-score plot and **F** volcano plot of comparison between the POI+hUCMSCs group and POI group show that the expression of most target metabolites was restored, manifesting as their levels being upregulated.

**Additional file 7: Supplementary figure 7.** Metabolomics analysis of the mechanisms of hUCMSCs treatment for POI. **A** Map of metabolic pathways of glycerolipid. **B** Pathway of cholesterol synthesis and steroid hormone biosynthesis. Significantly upregulated metabolites are shown in red, significantly downregulated metabolites are shown in blue, and nonsignificant different metabolites are shown in black. The names in bold are the relevant metabolites detected in the nontarget/target metabolomics analysis. The substance in the red ellipse represents the enzyme or its corresponding protein. The solid arrow indicates that metabolites (enzymes/proteins and metabolites) have a direct relationship with each other, while the dotted arrow indicates a distant relationship. Tissues or parts of metabolic processes (such as lysosomes) are represented by solid frames, and related pathways (such as glycerolipid pathways) are represented by dotted frames. The underlined metabolites represent the metabolites that recovered after hUCMSCs transplantation compared to the changes observed after POI induction. The green arrows represent the paths recovered.

**Additional file 8: Supplementary table 1.** Differential metabolomic changes induced by chemotherapy in ovarian tissue samples. (XLS 108 kb)

**Additional file 9: Supplementary table 2.** Differential metabolites screened by volcano map in serum samples after chemotherapy. (XLS 21 kb)

**Additional file 10: Supplementary table 3.** Differential lipid metabolomic changes in ovarian tissue samples of 3 groups (control, POI and POI+hUCMSCs). (XLS 314 kb)

**Additional file 11: Supplementary table 4.** Pseudotargeted metabolic differences of nonlipid metabolites in ovarian tissue samples of 3 groups (control, POI and POI+hUCMSCs). (XLS 292 kb)

## Data Availability

All data generated or analyzed during this study are included in this published article [and its supplementary information files].

## Abbreviations

POI: Premature ovarian insufficiency
MSCs: Mesenchymal stem cells
hUCMSCs: Human umbilical cord mesenchymal stem cells
HRT: Hormonal replacement treatment
PCOS: Polycystic ovary syndrome
CTX: Cyclophosphamide
BUS: Busulfan
H&E: Hematoxylin and eosin
UPLC: Ultra-performance liquid chromatography
MS: Mass spectrometry
QC: Quality control
ESI: Electrospray ionization
Arb: Arbitrary units
AGC: Automatic gain control
IT: Injection time
MRM: Multiple reaction monitoring
PRM: Parallel reaction monitoring
MTBE: Methyltert-butyl ether
LC-MS: Liquid chromatography-mass spectrometry
TIC: Total ion chromatogram
BPI: Basic peak ion chromatogram
RSDs: Relative standard deviations
GMP: Good manufacturing practice
OPLS-DA: Orthogonal partial least squares discrimination analysis
ISCT: International society for cellular therapy
PCA: Principal component analysis
