# Supplementary figures and images for: Human umbilical cord mesenchymal stem cells restore the ovarian metabolome and rescue premature ovarian insufficiency in mice
Source: Stem Cell Res Ther. 2020 Nov 4;11:466. doi: 10.1186/s13287-020-01972-5 (PMC7641864; doi:10.1186/s13287-020-01972-5)

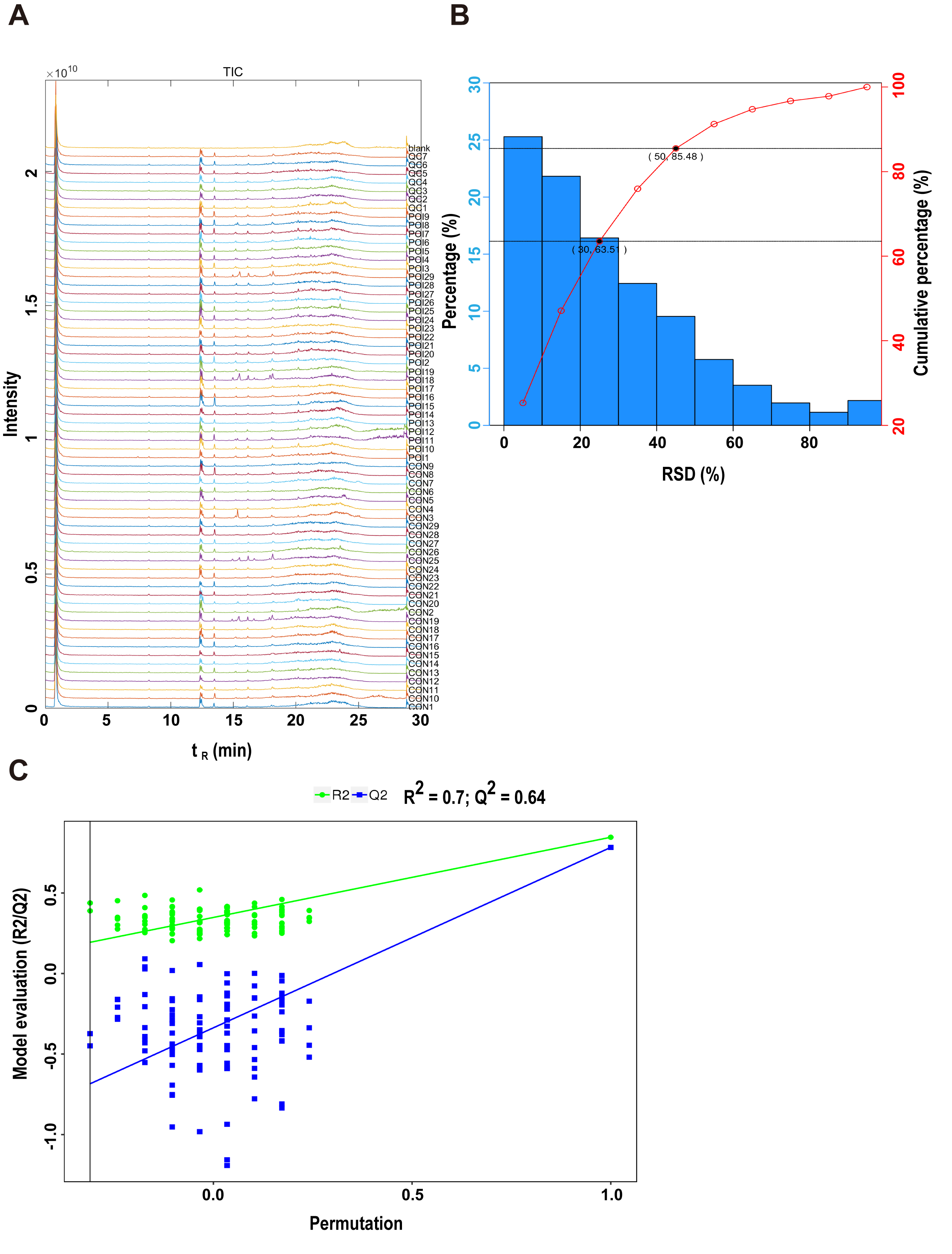

Supplement: Supplementary file 1 — Additional file 1: Supplementary figure 1. Metabolomics-data quality analysis of tissue samples from the control and POI modeling groups. A Total ion chromatograms (TICs) of all ovarian samples (the sum of the total number of ions and their time-varying curves for the various specific charges), which reflect the overall information of the samples. B Analysis of characteristic differences in quality control (QC) samples by metabolic mass spectrometry. The abscissa represents the relative standard deviation (RSD) of metabolic characteristics in QC samples, and the ordinate represents the percentage of metabolic characteristics whose RSD falls within the corresponding range. C Orthogonal partial least squares discriminatory analysis (OPLS-DA) was used to establish a model of the relationship between the expression of metabolites and samples, and the goodness of fit of the model could be predicted by the values of R2 and Q2. R2=0.70, Q2=0.64. [file 13287_2020_1972_MOESM1_ESM.tif]

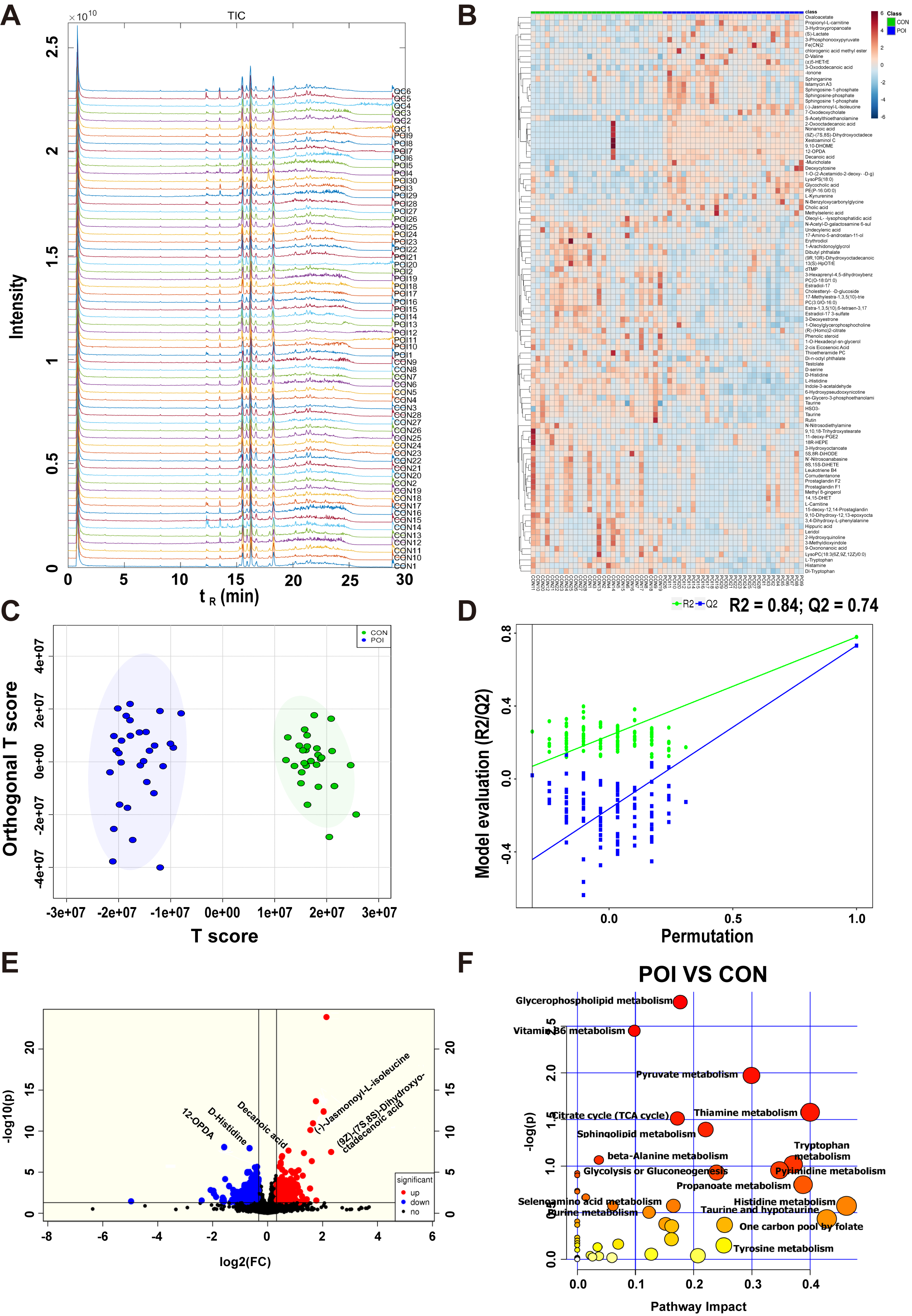

Supplement: Supplementary file 2 — Additional file 2: Supplementary figure 2. Metabolomics analysis of blood samples from the control group and POI model group. A Total ion chromatograms (TICs) of all blood samples, the sum of the total number of ions and their time-varying curves for the various specific charges, which reflect the overall information of the samples. B Heat map analysis of differential metabolome changes induced by chemotherapy in blood samples (control and POI groups). C OPLS-DA score plot of blood samples from both the control and POI groups. D Orthogonal partial least squares discriminatory analysis (OPLS-DA) was used to establish a model of the relationship between the expression of metabolites and blood samples, and the goodness of fit of the model could be predicted by the values of R2 and Q2. R2=0.84, Q2=0.74. E Volcano plot of screened differential metabolites in both groups. Each spot represents a metabolite, and the scattered spots represent the final screening result. Significantly upregulated metabolites are shown in red, significantly downregulated metabolites are shown in blue, and non-significantly different metabolites are shown in black. F KEGG was used to analyze the metabolic pathways in blood samples, and metabolic pathways with P < 0.05 are shown. [file 13287_2020_1972_MOESM2_ESM.tif]

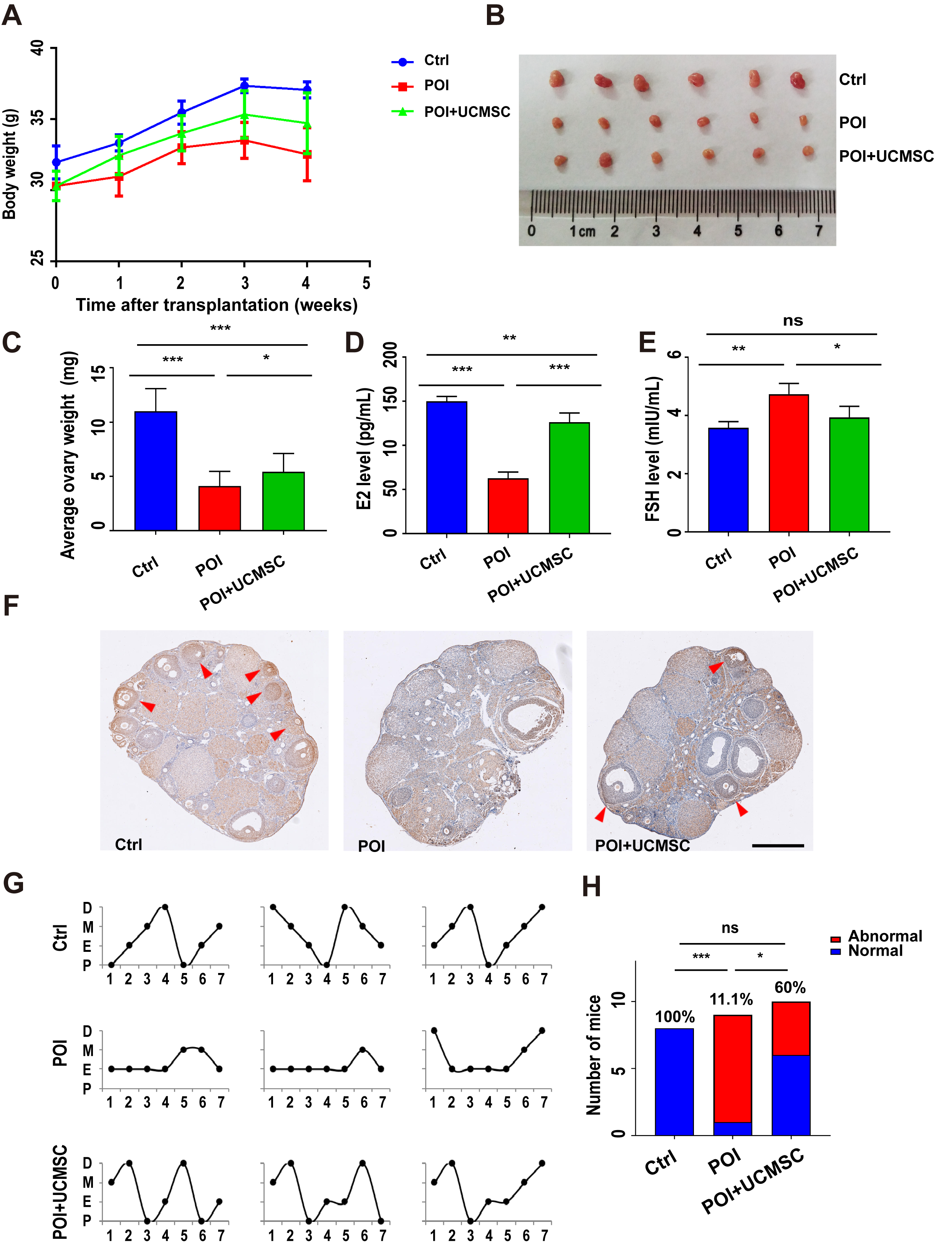

Supplement: Supplementary file 3 — Additional file 3: Supplementary figure 3. hUCMSCs transplantation promotes ovarian health status. A Line chart of body weight changes in the three groups of mice within 4 weeks after hUCMSCs transplantation (n=6, n=9, n=9). B Photograph of ovaries removed from the three groups after the hUCMSCs treatment. C The weights of the ovaries among the three groups were compared after the hUCMSCs treatment. D Measurement of serum E2 levels after hUCMSCs transplantation (n=4). E Measurement of serum FSH levels after hUCMSCs transplantation (n=4). F Immunohistochemical analysis of AMH expression (red arrowhead) in mouse ovaries. Scale bar = 500 μm. G The line charts are representative of estrous cycles in the three groups detected after the treatment cycle. D: diestrus, M: metestrus, E: estrus, P: proestrus. H The percentage of mice whose estrous cycles returned to normal after the treatment cycle (n=8, n=9, n=10). All data were presented as the mean ± SD. One-way ANOVA with LSD and Tamhane’s T2 post hoc test were used for analysis. *p < 0.05; **p < 0.01; ***p < 0.001. [file 13287_2020_1972_MOESM3_ESM.tif]

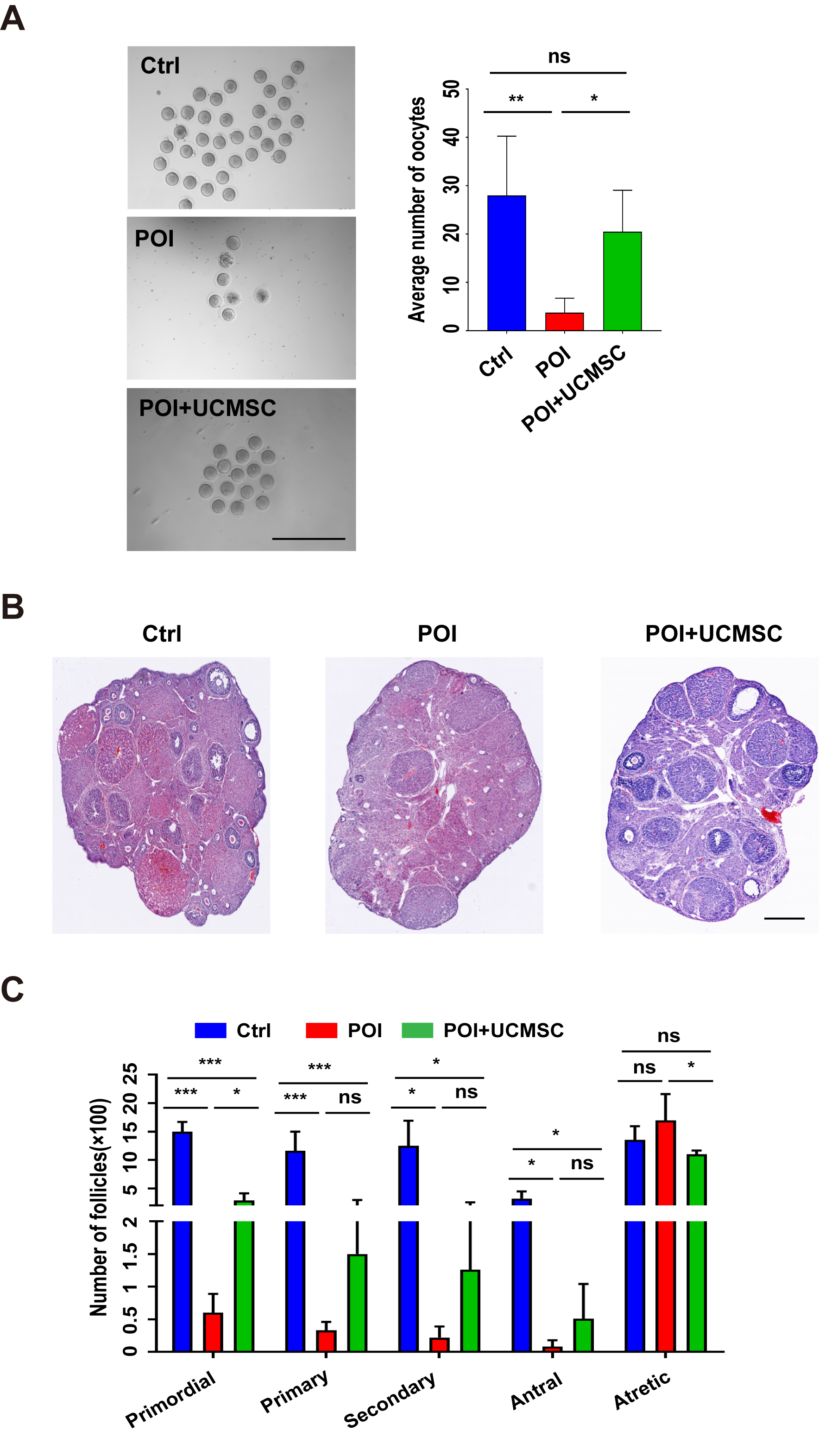

Supplement: Supplementary file 4 — Additional file 4: Supplementary figure 4. hUCMSCs transplantation promotes ovarian functional reserve. A Representative oocytes of three groups after superovulation. Scale bar = 400 μm. B H&E staining of ovarian morphological changes after hUCMSCs transplantation. Scale bar = 400 μm. C Quantitative analysis of different categories of ovarian follicles after hUCMSCs transplantation (n=5, n=6, n=5). All data were presented as the mean ± SD. One-way ANOVA with LSD and Tamhane’s T2 post hoc test were used for analysis. *p < 0.05; **p < 0.01; ***p < 0.001. [file 13287_2020_1972_MOESM4_ESM.tif]

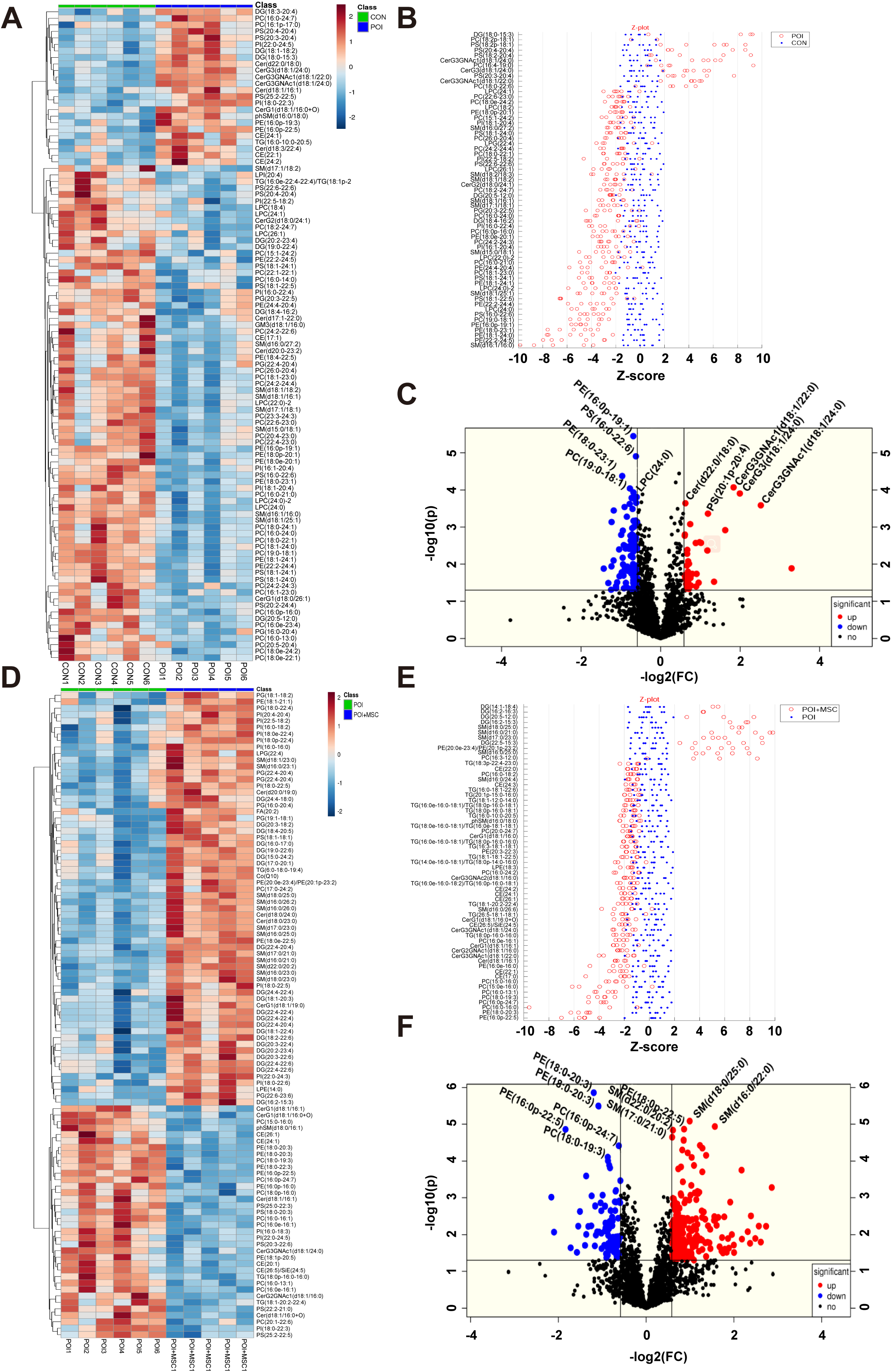

Supplement: Supplementary file 5 — Additional file 5: Supplementary figure 5. Nontarget metabolomics of lipids from ovarian samples. A Heat map showing that the expression levels of many lipid metabolites changed after POI modeling and that the patterns of the control and POI groups were significantly different. B Z-score plot of screened metabolites in the POI group with significant differences in the mean and standard deviation from that of the control group. Each spot represents a metabolite. The control group is shown in blue, and the POI group is shown in red. Z-scores were sorted in descending order, in which the magnitude of the change indicates the fold by which the mean of the control group is higher or lower than its standard deviation. C Volcano plot of the screened differential lipid metabolites measured in the control and POI groups. Each spot represents a metabolite, and the scattered spots represent the final screening result. Significantly upregulated metabolites are shown in red, significantly downregulated metabolites are shown in blue, and nonsignificant different metabolites are shown in black. D Heat map showing that the expression levels of a large number of metabolites from the POI+hUCMSCs group changed significantly compared with those from the POI group after transplantation. E Z-score plot of the metabolites screened in the samples of the POI+hUCMSCs group, with significant differences in the mean and standard deviation from those of the POI group. F Volcano plots of screened lipid metabolites measured in the POI and POI+hUCMSCs groups, with significant changes. [file 13287_2020_1972_MOESM5_ESM.tif]

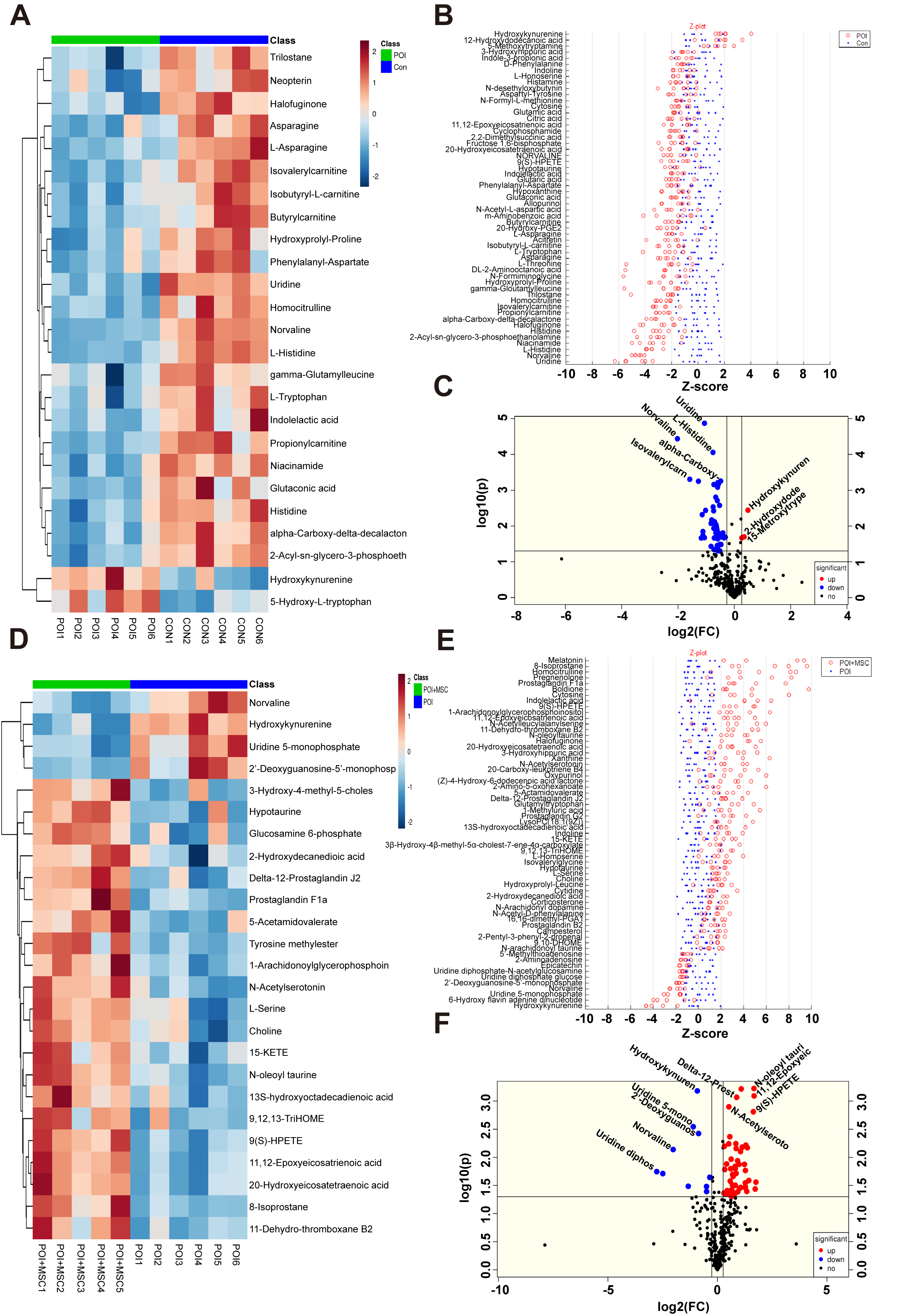

Supplement: Supplementary file 6 — Additional file 6: Supplementary figure 6. Pseudotargeted metabolomics of ovarian tissue samples. A Heat map results show that the expression levels of a large number of metabolites changed significantly after POI induction, and most decreased. B, C Z-score plot and volcano plot show that the expression levels of the metabolites unrelated to lipids were significantly changed after POI induction, and most decreased. D Heatmap, E z-score plot and F volcano plot of comparison between the POI+hUCMSCs group and POI group show that the expression of most target metabolites was restored, manifesting as their levels being upregulated. [file 13287_2020_1972_MOESM6_ESM.tif]

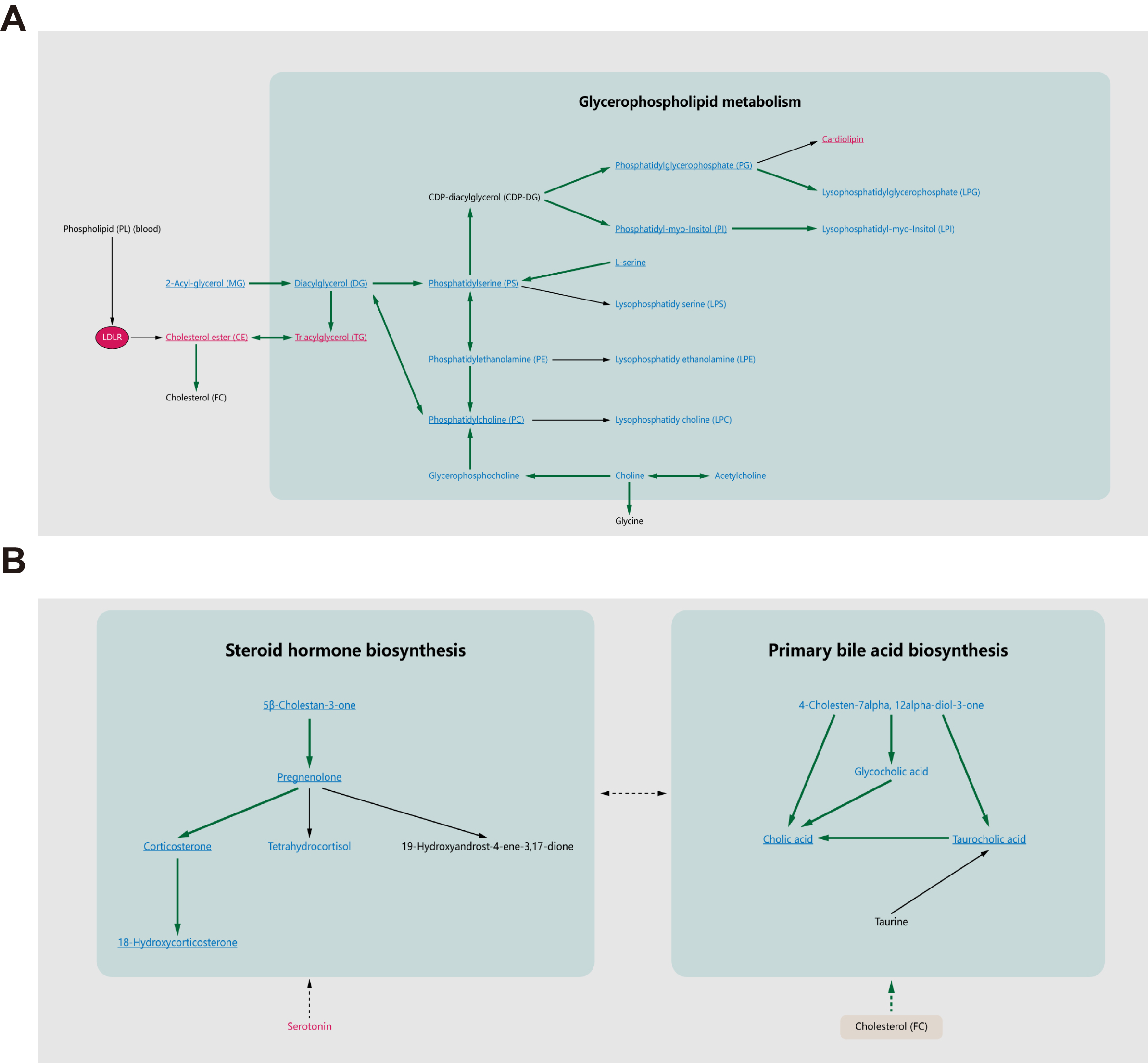

Supplement: Supplementary file 7 — Additional file 7: Supplementary figure 7. Metabolomics analysis of the mechanisms of hUCMSCs treatment for POI. A Map of metabolic pathways of glycerolipid. B Pathway of cholesterol synthesis and steroid hormone biosynthesis. Significantly upregulated metabolites are shown in red, significantly downregulated metabolites are shown in blue, and nonsignificant different metabolites are shown in black. The names in bold are the relevant metabolites detected in the nontarget/target metabolomics analysis. The substance in the red ellipse represents the enzyme or its corresponding protein. The solid arrow indicates that metabolites (enzymes/proteins and metabolites) have a direct relationship with each other, while the dotted arrow indicates a distant relationship. Tissues or parts of metabolic processes (such as lysosomes) are represented by solid frames, and related pathways (such as glycerolipid pathways) are represented by dotted frames. The underlined metabolites represent the metabolites that recovered after hUCMSCs transplantation compared to the changes observed after POI induction. The green arrows represent the paths recovered. [file 13287_2020_1972_MOESM7_ESM.tif]
